# Comparative Analysis of the Peanut Witches'-Broom Phytoplasma Genome Reveals Horizontal Transfer of Potential Mobile Units and Effectors

**DOI:** 10.1371/journal.pone.0062770

**Published:** 2013-04-23

**Authors:** Wan-Chia Chung, Ling-Ling Chen, Wen-Sui Lo, Chan-Pin Lin, Chih-Horng Kuo

**Affiliations:** 1 Institute of Plant and Microbial Biology, Academia Sinica, Taipei, Taiwan; 2 Molecular and Biological Agricultural Sciences Program, Taiwan International Graduate Program, National Chung Hsing University and Academia Sinica, Taipei, Taiwan; 3 Graduate Institute of Biotechnology, National Chung Hsing University, Taichung, Taiwan; 4 Department of Plant Pathology and Microbiology, National Taiwan University, Taipei, Taiwan; University of Lausanne, Switzerland

## Abstract

Phytoplasmas are a group of bacteria that are associated with hundreds of plant diseases. Due to their economical importance and the difficulties involved in the experimental study of these obligate pathogens, genome sequencing and comparative analysis have been utilized as powerful tools to understand phytoplasma biology. To date four complete phytoplasma genome sequences have been published. However, these four strains represent limited phylogenetic diversity. In this study, we report the shotgun sequencing and evolutionary analysis of a peanut witches'-broom (PnWB) phytoplasma genome. The availability of this genome provides the first representative of the 16SrII group and substantially improves the taxon sampling to investigate genome evolution. The draft genome assembly contains 13 chromosomal contigs with a total size of 562,473 bp, covering ∼90% of the chromosome. Additionally, a complete plasmid sequence is included. Comparisons among the five available phytoplasma genomes reveal the differentiations in gene content and metabolic capacity. Notably, phylogenetic inferences of the potential mobile units (PMUs) in these genomes indicate that horizontal transfer may have occurred between divergent phytoplasma lineages. Because many effectors are associated with PMUs, the horizontal transfer of these transposon-like elements can contribute to the adaptation and diversification of these pathogens. In summary, the findings from this study highlight the importance of improving taxon sampling when investigating genome evolution. Moreover, the currently available sequences are inadequate to fully characterize the pan-genome of phytoplasmas. Future genome sequencing efforts to expand phylogenetic diversity are essential in improving our understanding of phytoplasma evolution.

## Introduction

Phytoplasmas are a group of phytopathogenic bacteria that are transmitted by sap-feeding insect vectors [1]–[3]. Phylogenetically, phytoplasmas are related to the animal pathogenic mycoplasmas. Both groups are unique among bacteria in their lack of cell wall and are assigned to the class Mollicutes. However, unlike mycoplasmas that can be cultured and are amenable to genetic manipulations, the *in vitro* cultivation of phytoplasmas has remained unsuccessful despite decades of effort [4]. The inability to culture phytoplasmas outside of their hosts has resulted in the designation of the ‘*Candidatus* (*Ca*.)’ status in their taxonomic assignment [2] and also greatly hampered the research progresses in characterizing these plant pathogens.

With the recent advancements in genomic sciences, genome sequencing has been adopted as a powerful tool to characterize the gene content of these uncultivated bacteria [5]–[9]. More importantly, comparative analyses of the available phytoplasma genome sequences have improved our knowledge of their evolutionary history and facilitated experimental works to investigate the mechanisms of pathogenicity [10]–[13]. In this study, we performed whole genome shotgun sequencing of a peanut witches'-broom (PnWB) phytoplasma. The availability of this genome sequence substantially improves the taxon sampling for the characterization of genome diversity in phytoplasmas. Furthermore, comparative analysis with previously sequenced phytoplasmas provides insights into the genome evolution in these bacteria.

## Results and Discussion

### Phylogenetic placement of the PnWB phytoplasma

**Figure 1:**
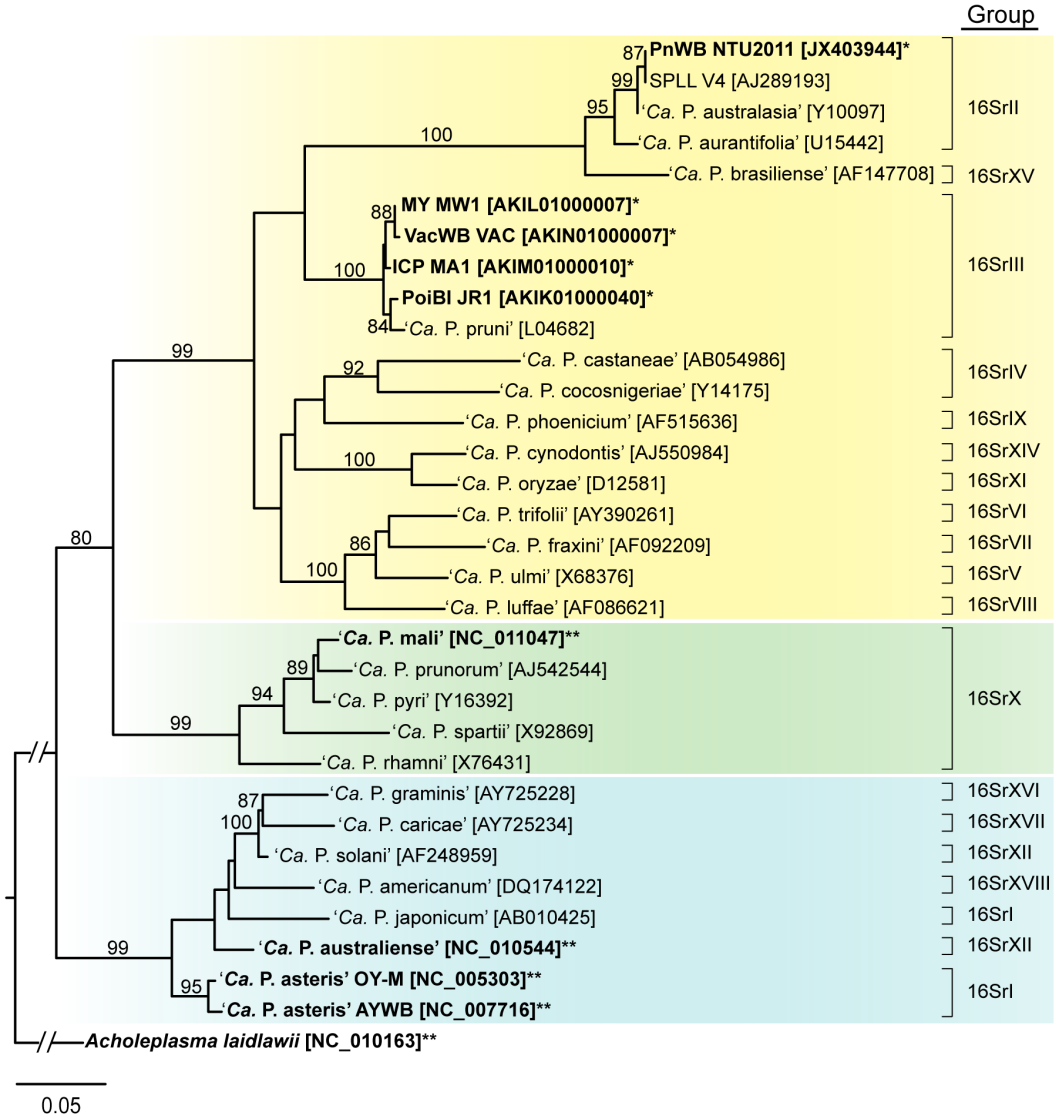
Molecular phylogeny of phytoplasmas. The maximum likelihood tree is inferred based on the 16S ribosomal RNA gene. GenBank accession numbers are listed in square brackets following the species names. The numbers on the internal branches indicate the percentage of bootstrap support based on 10,000 re-sampling (only values >80% are shown). The sequence from *Acholeplasma laidlawii* is included as the outgroup. The species with genome sequences available are highlighted in bold (*partial; **complete). Abbreviations: peanut witches'-broom phytoplasma strain NTU2011 (PnWB NTU2011); sweet potato little leaf phytoplasma strain V4 (SPLL V4); milkweed yellows phytoplasma strain MW1 (MY MW1); Vaccinium witches'-broom phytoplasma strain VAC (VacWB VAC); Italian clover phyllody phytoplasma strain MA1 (ICP MA1); poinsettia branch-inducing phytoplasma strain JR1 (PoiBI JR1).

According to the molecular phylogeny inferred from the 16S ribosomal RNA genes, the genus ‘*Ca.* Phytoplasma’ contains three major clades (Figure 1). The first and the most basal clade (highlighted by a blue background in Figure 1) contains groups such as aster yellows (16SrI), stolbur (16SrXII), sugarcane yellow leaf syndrome (16SrXVI), papaya bunchy top (16SrXVII), and American potato purple top wilt (16SrXVIII). The second clade (highlighted by a green background in Figure 1) is mainly composed of the apple proliferation group (16SrX). The third clade (highlighted by a yellow background in Figure 1) is the most diverse one and contains the highest number of 16S rRNA groups [1], [2]. Prior to this study, three of the four published phytoplasma genomes belong to the first clade, including two strains of ‘*Ca*. P. asteris’ [5], [6] in the aster yellows group (16SrI) and one strain of ‘*Ca*. P. australiense’ [7] in the stolbur group (16SrXII). The remaining one is a ‘*Ca*. P. mali’ [8] in the apple proliferation group (16SrX) from the second clade. Although four draft genomes from the 16SrIII group have been published recently [9], these assemblies are highly fragmented (each containing 158–272 contigs with a median length of ∼0.7–1.7 kb) and do not have annotation yet (as of November, 2012; GenBank accession numbers AKIK00000000.1, AKIL00000000.1, AKIM00000000.1, and AKIN00000000.1). Thus, the PnWB phytoplasma reported in this study is the first representative of the 16SrII group and provides a high quality draft for the third clade.

Within the third clade, the PnWB phytoplasma belongs to the peanut witches'-broom group (16SrII). This organism was initially collected from naturally infected peanut plants in Taiwan and is closely related to the sweet potato little leaf (SPLL) phytoplasma and ‘*Ca*. P. australasia’ that was associated with papaya yellow crinkle [14]. Both the SPLL phytoplasma and ‘*Ca*. P. australasia’ were collected in Australia, suggesting that these phytoplasmas may have originated from the Southeast Asia and Oceania region.

### PnWB phytoplasma genome assembly

The draft genome assembly of the PnWB phytoplasma strain NTU2011 contains 13 chromosomal contigs with a total size of 562,473 bp (Table 1). Despite the utilization of two Illumina libraries and extensive efforts devoted to gap closing, we were not able to sequence the genome to completion. Because no method exists for *in vitro* cultivation of this obligate pathogen, host DNA contaminations, particularly those from the chloroplast genome, introduced a high level of noise in the assembly process. Furthermore, a large number of repetitive elements (size range  = 30–300 bp) exist in the PnWB phytoplasma genome, which complicated the use of short Illumina reads for *de novo* assembly. Finally, the PnWB phytoplasma genome has a very low GC content (genome average  = 24.3%; see Table 1). This strong nucleotide composition bias is associated with the abundance of long homopolymers and low complexity sequences, which resulted in several unresolvable problems in gap filling using PCR and Sanger sequencing.

**Table 1:** Genome assembly statistics.

Genome	PnWB NTU2011	‘*Ca.* P. mali’	‘*Ca.* P. australiense’	‘*Ca.* P. asteris’ AYWB	‘*Ca.* P. asteris’ OY-M
Accession	AMWZ00000000	NC_011047	NC_010544	NC_007716	NC_005303
No. of contigs	13	1	1	1	1
Length (bp)	562,473	601,943	879,959	706,569	853,092
G+C content (%)	24.3	21.4	27.4	26.9	27.8
No. of protein-coding genes	421	479	684	671	749
Coding density (%)	68.2	76.1	64.1	73.5	72.8
No. of tRNA genes	27	32	35	31	32
No. of rRNA operons	2	2	2	2	2

Our attempts to estimate the genome size by using pulsed-field gel electrophoresis (PFGE) were unsuccessful (data not shown), possibly due to the low abundance of this phytoplasma in the periwinkle host. The closely related SPLL phytoplasma has an estimated genome size of 622 kb [15]. Using this size as a reference, our draft assembly was estimated to cover ∼90% of the PnWB phytoplasma genome. To provide an alternative method of estimating the completeness, we identified a list of protein-coding genes that are conserved among the four complete phytoplasma genomes (Table 1) and checked for their presence in our draft assembly. This approach produced a similar estimate of ∼89% completeness.

In addition to the chromosomal contigs, our assembly also contains one complete plasmid sequence. This circular plasmid is 4,221 bp in size and encodes four proteins (replication protein, putative DNA primase, threonine synthase, and hypothetical protein). This PnWB phytoplasma whole genome shotgun project has been deposited at DDBJ/EMBL/GenBank under the accession AMWZ00000000 (BioProject PRJNA89719). The version described in this paper is the first version, AMWZ01000000.

**Figure 2:**
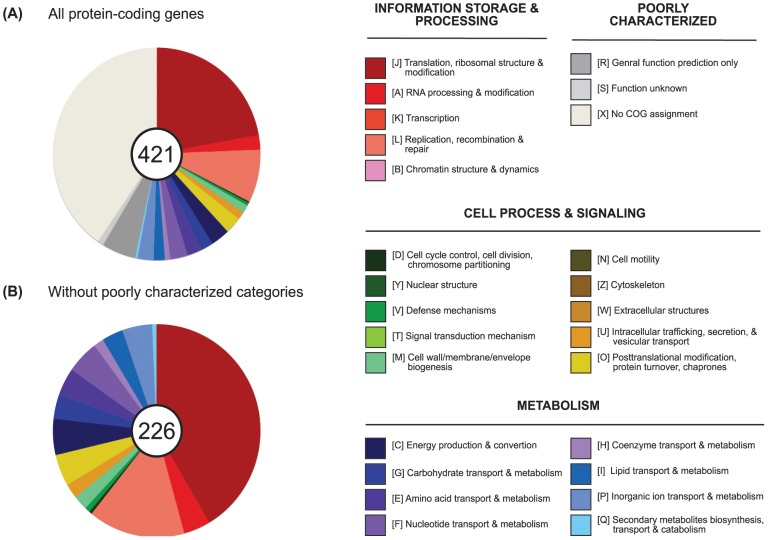
Functional classification of annotated protein-coding genes according to COG assignments. Genes without inferred COG annotation were assigned to a custom category X. The number of protein-coding genes in each set is labeled in the center of each pie chart. (A) All 421 annotated protein-coding genes in the PnWB NTU2011 genome. (B) The 226 protein-coding genes that have specific functional category assignments.

The number of protein-coding genes in the PnWB phytoplasma is similar to ‘*Ca*. P. mali’ and relatively low compared to ‘*Ca*. P. asteris’ and ‘*Ca*. P. australiense’ (Table 1). Only ∼53% of the proteins can be assigned to COG categories [16], [17] with specific functions (Figure 2A). This estimate is similar to that of ‘*Ca*. P. mali’ (54%) and higher than those of ‘*Ca*. P. asteris’ and ‘*Ca*. P. australiense’ (39–42%), which reflects the fact that phytoplasmas are highly divergent from model organisms and lack empirical studies. Among the proteins that lack specific COG assignments, 81 (19% of total) were annotated as hypothetical proteins. This proportion of proteins lacking specific description is typical among currently available bacterial genomes. However, a sizable portion of proteins that lack COG assignments do have specific functional annotation. For example, the PnWB phytoplasma genome encodes for 48 putative effectors (assigned to 42 homologous gene clusters; accounting for 11% of total). These putative effectors are similar to known pathogenicity factors of phytoplasmas (see below) but are not included in the COG database. Additionally, several putative transporters (e.g., *citS*, *dppB*, *dppD*, *dppF*, *nlpA*, etc) could be annotated on the basis of sequence similarity but lack COG assignments.

Among the 226 proteins with specific COG category assignments, translation (COG category J) and DNA replication (COG category L) represent the two most abundant categories (Figure 2B). Inspection of gene content reveals that the PnWB phytoplasma has limited metabolic capacity (see below). Among the proteins that are assigned to COG metabolism categories, 24 out of 67 (∼36%) are related to transporters, indicating the high reliance of this obligate pathogen to import nutrients from its hosts.

The largest gene family in the PnWB phytoplasma genome is a group of retron-type reverse transcriptases, including two full-length coding sequences and 12 disrupted pseudogenes. These genes have homologous sequences in ‘*Ca*. P. asteris’ and ‘*Ca*. P. australiense’. The presence of these genes across diverse phytoplasma lineages suggests that they existed in the ancestral phytoplasma genome, which is consistent with the hypothesis that recurrent phage attacks shaped the early genome evolution in phytoplasma [18].

### Comparative analysis of gene content

**Figure 3:**
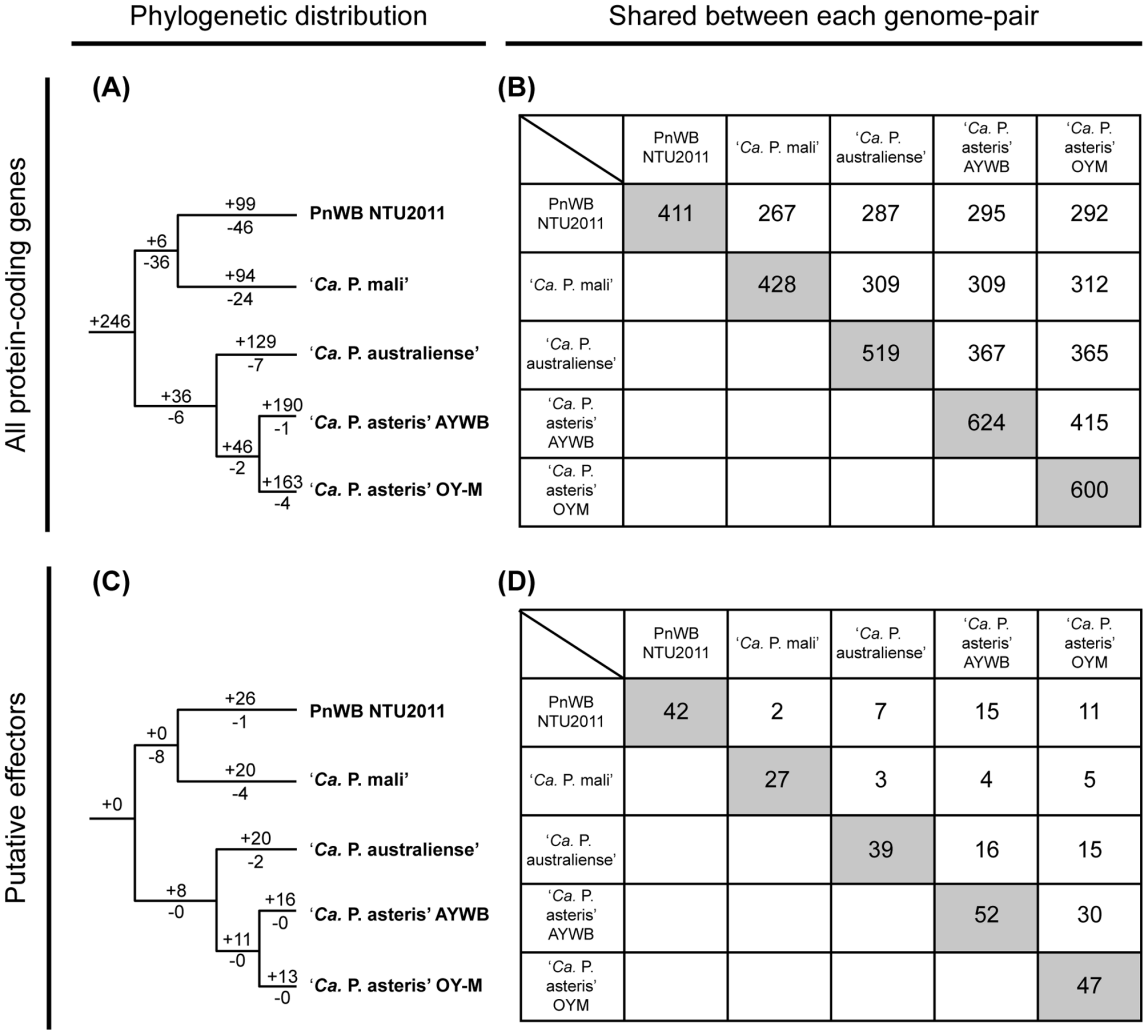
Distribution patterns of homologous gene clusters. Panels (A) and (B) are based on all protein-coding genes, panels (C) and (D) are based on the putative effectors. Panels (A) and (C) illustrate the phylogenetic distribution patterns of homologous gene clusters. The numbers above a branch and preceded by a ‘+’ sign indicate the number of clusters that are uniquely present in all daughter lineages, the numbers below a branch and preceded by a ‘−’ sign indicate the number of clusters that are uniquely absent. For example, in panel (A) PnWB NTU2011 and ‘*Ca. P.* mali’ share six homologous gene clusters that are not found in any of the other three phytoplasma genomes; similarly, 36 clusters are absent in both PnWB NTU2011 and ‘*Ca.* P. mali’ but are shared by the other three phytoplasma genomes. Panels (B) and (D) illustrate the number of homologous gene clusters shared between each genome-pair, numbers on the diagonal indicate the number of clusters found in each individual genome.

**Figure 4:**
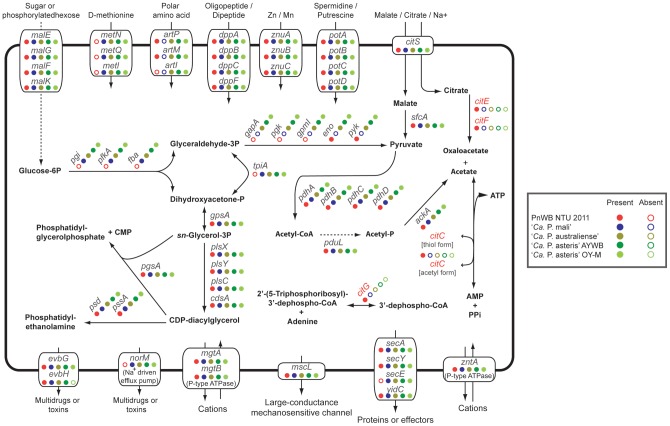
Highlights of selected metabolic pathways and transporters in phytoplasmas. The color-coded circles indicate the presence (filled) or the absence (unfilled) of a gene in each genome. Genes that are present in the PnWB phytoplasma and absent in the other four genomes are highlighted in red.

The comparative analysis of the five phytoplasma genomes (Table 1) reveals a conserved core of 246 homologous gene clusters shared by all lineages surveyed (Figure 3A; Tables S1 and S2; see Materials and Methods for the definition of gene clusters). Approximately half of these conserved genes belong to the information storage and processing COG categories, such as DNA replication, transcription, and translation. The most dominant metabolic categories are nucleotide metabolism (*adk*, *cmk*, *dut*, *gmk*, *hit*, *nrdE*, *nrdF*, *pyrG*, *pyrH*, *tdk*, *thyA*, and *tmk*), energy production (*ackA*, *evbG*, *gpsA*, *iscU*, *pdhA*, *pdhB*, *pdhC*, *pdhD*, *ppa*, and *sfcA*), inorganic ion metabolism (*cbiO*, *cbiQ*, *dppC*, *mgtA*, *mgtB*, *sodA*, *znuA*, *znuB*, and *znuC*), and lipid metabolism (*acpP*, *acpS*, *cdsA*, *pgsA*, *plsC*, *plsX*, *psd*, and *pssA*) (Figure 4; Tables S1 and S2).

Due to the incompleteness of the PnWB phytoplasma genome assembly, this list of conserved genes may have been underestimated. For example, although several known genes in the glycolysis pathway (*pgi*, *pfkA*, *fba*, and *tpiA*) and transporters (*metN*, *metQ*, and *metI*) were absent in the current PnWB phytoplasma genome annotation (Figure 4), they may be located in the unassembled regions of this genome. It is therefore difficult to conclude whether the glycolysis pathway is complete in the PnWB phytoplasma. A total of 29 putative pseudogenes with disrupted open reading were identified in our first version of annotation (e.g., *gpmI*, *mesJ*, *metK*, *polA*, *trmE*, etc). It is unclear if functional copies of these genes exist in the unassembled regions or not.

Notably, genome-specific genes account for ∼22–30% of gene content (Figures 3A and 3B). These estimates are quite high considering that two closely related strains of ‘*Ca*. P. asteris’ are included in the comparison. This finding suggests that acquisition of novel genes is important in the diversification of phytoplasmas and may contribute to their adaptation to different ecological niches. For example, the PnWB phytoplasma genome contains several genes related to citrate fermentation (i.e., *citE*, *citF*, *citC*, and *citG*; see Figure 4) that are not found in the other four phytoplasmas compared. Considering that citrate and malate are the predominant organic acids in phloem sap, the ability to utilize citrate may provide more versatile metabolism for this obligate pathogen. However, because most of the genes involved in the TCA cycle are not found in the PnWB phytoplasma genome (as well as the other four), it is unlikely that this pathogen can rely on the TCA cycle for energy production. Rather, the uptake and utilization of citrate and malate may provide the major sources for carbohydrate metabolism.

In addition to the metabolic genes, a large fraction of putative effectors are genome-specific (Figures 3C and 3D; Table S3). These secreted proteins are utilized by phytoplasmas to manipulate their plant hosts and can alter the interactions between the plant hosts and insect vectors [19]–[22]. The pattern that a large fraction of the putative effectors appear to be species- or strain-specific indicates that these effectors tend to evolve faster than the other genes in the phytoplasma genomes and are likely to be important in lineage-specific adaptation to different hosts.

**Figure 5:**
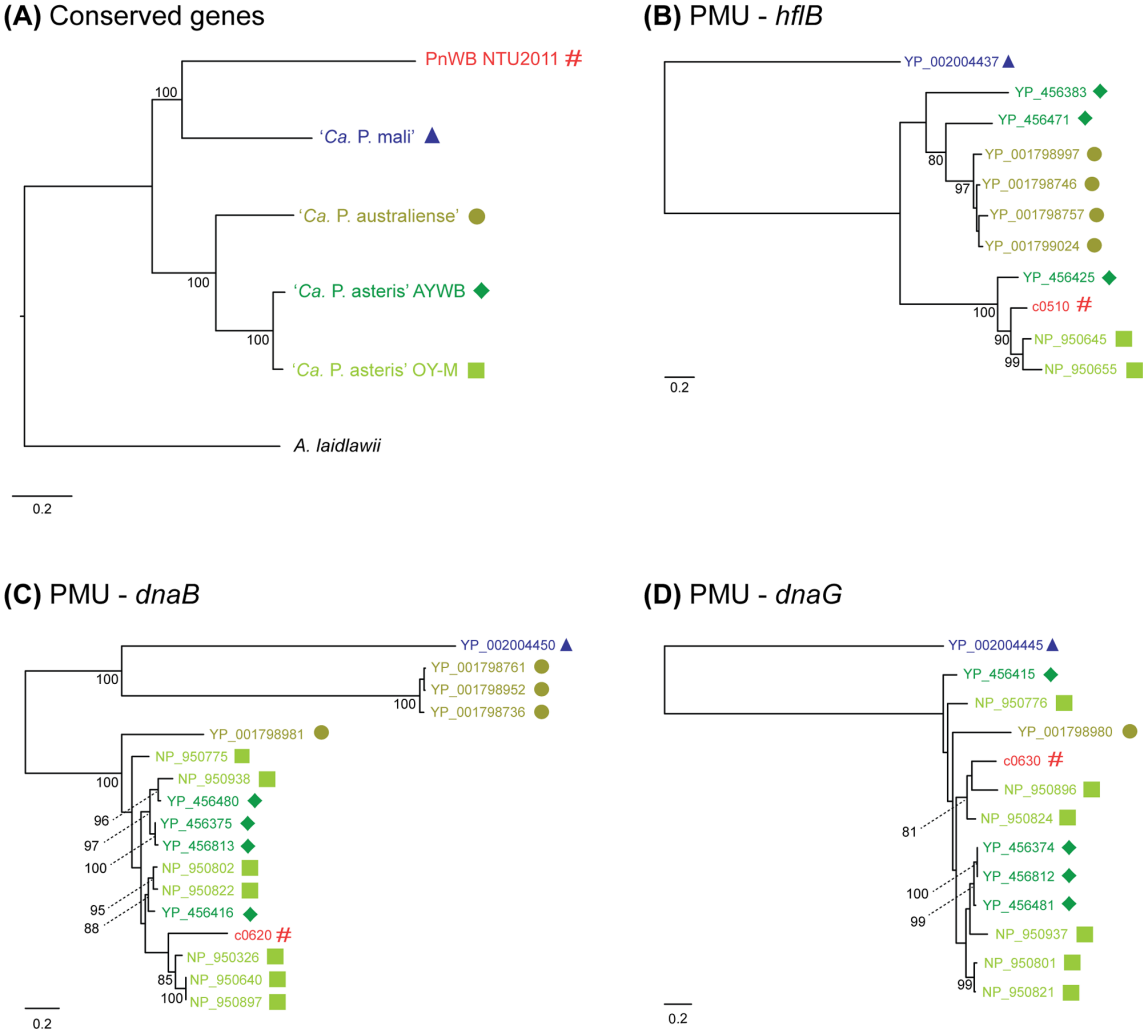
Molecular phylogeny of conserved and PMU-associated genes. The numbers on the internal branches indicate the percentage of bootstrap support based on 1,000 re-sampling (only values >80% are shown). (A) The organismal phylogeny based on the concatenated alignment of 214 single-copy genes conserved among the five phytoplasma genomes and the outgroup *A. laidlawii* (75,565 aligned amino acid sites). The tree topology is consistent with that inferred from 16S rRNA genes (Figure 1). (B) Molecular phylogeny of PMU-associated ATP-dependent Zn protease (*hflB*; 855 aligned amino acid sites). (C) Molecular phylogeny of PMU-associated replicative DNA helicase (*dnaB*; 533 aligned amino acid sites). (D) Molecular phylogeny of PMU-associated DNA primase (*dnaG*; 614 aligned amino acid sites). Note that the outgroup is not included in panels (B)-(D) because no PMU was found in the *A. laidlawii* genome.

Examination of the gene distribution patterns revealed two interesting points regarding the genome evolution in phytoplasmas. Based on the species tree inferred from the 16S rRNA (Figure 1) and conserved protein-coding genes (Figure 5A), the PnWB phytoplasma and ‘*Ca*. P. mali’ share a more recent common ancestor than with ‘*Ca*. P. asteris’. If the evolution of gene content followed the species phylogeny, we expect to see that these two genomes would share more homologous gene clusters with each other than with the more distantly related ‘*Ca*. P. asteris’. However, the opposite pattern was found for all protein-coding genes (Figure 3B). The comparison of genome sizes, coding density (Table 1), and the phylogenetic distribution pattern of genes (Figure 3A) suggest that this observation can be explained by extensive independent gene losses in these two lineages with highly reduced genomes.

The second intriguing finding is that the PnWB phytoplasma shares a high proportion of its putative effectors with ‘*Ca*. P. asteris’, while the ‘*Ca*. P. mali’ effectors are mostly genome-specific (Figure 3D). Unlike the pattern for all protein-coding genes (Figure 3C), independent losses of ancestral effectors in the PnWB phytoplasma and ‘*Ca*. P. mali’ appeared to be a less likely explanation. Rather, transposon-mediated horizontal transfer of effectors between the PnWB phytoplasma and ‘*Ca*. P. asteris’ provides a more plausible hypothesis (see below).

### Molecular evolution of potential mobile units

One interesting feature of the phytoplasma genomes is the presence of potential mobile units (PMUs). These putative transposons are linked to the genome instability observed in these bacteria [6], [7], [10], [18], [23]. More importantly, several experimentally characterized effectors are associated with such PMUs [13], suggesting that these mobile units may contribute to the evolution of phytopathogenicity. Our draft assembly of the PnWB phytoplasma genome contains one PMU (Table S4). Although it is possible that additional copies exist in the unassembled regions, the raw read coverage patterns for this PMU from both sequenced libraries are similar to those for single-copy genes (*e.g.*, *dnaN*), suggesting that this PMU is likely to be single-copy as well.

The molecular phylogenies of the three longest PMU-associated genes support the hypothesis for horizontal transfer of these putative transposons. All three gene trees (*hflB*, *dnaB*, and *dnaG*) indicate that the PnWB PMU is similar to those in the two ‘*Ca*. P. asteris’ genomes, while the ‘*Ca*. P. mali’ PMU is highly divergent (Figures 5B, 5C, and 5D). The ‘*Ca*. P. australiense’ PMUs show a more complex pattern because five types of PMUs exist in this genome and these PMUs can be divided into two classes based on gene composition [7]. Examination of gene order provides further support to the molecular phylogeny results (Table S4). The PnWB PMU has similar structure to those in ‘*Ca*. P asteris’ [6], while the homologous genes in the ‘*Ca*. P. mali’ PMU [8] have a different arrangement (e.g., the *dnaB* and *dnaG* are separated by four hypothetical proteins instead of being adjacent to each other). The PMU5 in ‘*Ca*. P. australiense’ is similar to those in ‘*Ca*. P. asteris’ while the PMU1-4 are more similar to each other and appear to have a different origin.

The observation that PMUs may have been horizontally transferred among divergent phytoplasma lineages has important implications for the evolution of phytopathogenicity. The point that horizontal gene transfer may be important in the phytoplasma genome evolution has been proposed previously [9]. Through molecular phylogenetic inference of PMU-related genes and comparative analysis of gene orders in PMUs, this study further establishes that gene transfers among phytoplasmas may be facilitated by these transposon-like elements. Given the tight association between effectors and PMUs [13], [19], the horizontal transfer of PMUs can provide these pathogens with novel weaponry against their hosts and contribute to their adaptation.

In summary, the PnWB phytoplasma genome sequence from this study provides the first representative of a major group (i.e., 16SrII) that was previously uncharacterized at genome-scale. Despite the fact that this genome was not sequenced to completion, the current assembly covers ∼90% of the genome. This high quality draft can serve as a reference for gene cloning to facilitate functional studies of this uncultivated bacterium. The comparative analysis of gene content among available phytoplasma genomes indicates that the 22–30% of the genes appear to be genome-specific (Figure 3A). This finding suggests that the pan-genome of these phytopathogenic bacteria is quite large and future improvements in taxon sampling (particularly for the 16S groups such as 16SrIV-16SrIX and 16SrXIV-16SrXVIII; see Figure 1) are required to further investigate their genome diversity. Additionally, PMU-mediated horizontal gene transfers among divergent phytoplasmas are likely to be an important factor in the evolution of pathogenicity.

## Materials and Methods

### Strain source and DNA preparation

The PnWB phytoplasma was initially collected from naturally infected peanut plants on the Penghu Islands of Taiwan in 1975 (I.-L. Yang, Taiwan Agricultural Research Institute). This phytoplasma was transferred to periwinkle (*Catharanthus roseus*) through dodder (*Cuscuta australis*) and maintained by side grafting in the green house at the National Taiwan University. The strain PnWB NTU2011 was collected from artificially infected periwinkle in July, 2011. For DNA extraction, 1.4 g of midribs from mature leaves were grounded using a ceramic mortar and pestle set with liquid nitrogen. The Wizard Genomic DNA Purification Kit (Promega) was used for total DNA purification according to the manufacturer's protocol.

### Molecular phylogenetic inference

To determine the phylogenetic placement of the PnWB phytoplasma, the partial 16S ribosomal RNA gene was amplified by using the PCR primer pair P1 (5′-AAG-AGT-TTG-ATC-CTG-GCT-CAG-GAT-T-3′) and P7 (5′-CGT-CCT-TCA-TCG-GCT-CTT-3′). Each 50 ul reaction mix contained 25 ul of DreamTaq Master Mix (Fermentas), 0.2 uM of each primer, and 125 ng of DNA template. The cycling conditions were: (1) an initial activation step at 95°C for 5 min; (2) 35 cycles of 95°C for 30 sec, 53°C for 1 min, and 72°C for 1.5 min; and (3) a final extension step for 10 min. The PCR product of expected size was purified using the Illustra GFX PCR Gel Band Purification Kit (GE Healthcare Life Sciences) and sequenced using the BigDye Terminator v3.1 Cycle Sequencing Kit on an ABI Prism 3700 Genetic Analyzer (Applied Biosystems).

This partial 16S rRNA gene sequence was deposited at the DDBJ/EMBL/GenBank under the accession JX403944. A set of 16S rRNA gene sequences from other phytoplasmas and the outgroup *Acholeplasma laidlawii* PG-8A were downloaded from GenBank for molecular phylogenetic inference (see Figure 1 for accession numbers). These sequences were aligned using MUSCLE [24] with the default settings. The resulting multiple sequence alignment was used to infer the species phylogeny using the maximum likelihood program PhyML [25]. The transition/transversion ratio, proportion of invariable sites, and the gamma distribution parameter (with four categories of substitution rates) were estimated from the alignment in the maximum likelihood framework. To estimate the level of support for each internal branch, we generated 10,000 non-parametric bootstrap samples of the alignment by using the SEQBOOT program in the PHYLIP package [26] and repeated the phylogenetic inference as described above.

### Shotgun sequencing and genome assembly

To obtain the genome sequence of PnWB NTU2011, we used a commercial service provider (Yourgene Bioscience, Taipei, Taiwan) for shotgun sequencing based on the Illumina HiSeq 2000 platform (Illumina). Two separate libraries were prepared and sequenced, including one paired-end library (insert size  = ∼223 bp, 149,717,490 read-pairs, ∼30.2 Gb of raw data) and one mate-pair library (insert size  = ∼4.5 kb, 13,233,069 read-pairs, ∼2.7 Gb of raw data).

The *de novo* genome assembly was initially based on the paired-end reads because of their low variation in insert size. The procedure for the assembly and annotation was largely based on that described in one of our previous studies [27]. The raw reads were trimmed at the first position from the 5′-end that has a quality score of lower than 20. After discarding reads that were shorter than 50 bp, we obtained a high quality set of 165,464,660 paired and 28,543,039 unpaired reads (∼17.7 Gb of raw data). These reads were used as the input for Velvet version 1.1.06 [28] with the following settings: k = 53, exp_cov  = 15,000, cov_cutoff  = 60, max_coverage  = 60,000, and min_contig_lgth  = 500. The high expected coverage setting was chosen based on the actual coverage pattern observed in our assembly parameter tests.

To identify putative phytoplasma contigs in the initial draft assembly, we performed BLASTX [29], [30] searches against a custom database that contained all 8,928 ‘*Candidatus* Phytoplasma’ (Taxonomy ID 33926) protein sequences in GenBank [31] as of November, 2011. Contigs with a hit with an e-value of lower than 1e^−5^ were selected for manual inspection to further remove possible contaminations from the plant host. The remaining contigs were used as the starting point for our iterative assembly improvement process. For each iteration, we mapped all raw reads from the paired-end and the mate-pair libraries to the contigs using BWA [32] and visualized the result using IGV [33]. Neighboring contigs with mate-pair support for continuity were merged into scaffolds and polymorphic sites were examined using the MPILEUP program in the SAMTOOLS package [34]. For regions that appeared to have been mis-assembled, which were often associated with aberrant coverage patterns, we extracted the raw reads that were mapped to the corresponding regions and re-assembled these regions by PHRAP (http://www.phrap.org/). In addition, PCR and Sanger sequencing were used to fill gaps and to verify the correctness of the assembly. These updates were incorporated into the assembly and examined in the next iteration. We repeated this iterative process until no improvement could be made with the method described.

### Genome annotation and homologous gene identification

To annotate the PnWB NTU2011 genome, we utilized RNAmmer [35], tRNAscan-SE [36], and Prodigal [37] to predict the locations of rRNA, tRNA, and protein-coding genes. The four complete phytoplasma genomes available (Table 1) were used as the primary references to assign gene names and description of protein-coding genes. The homologous gene identification was based on the procedure developed in our previous study [11]. Briefly, we performed all-against-all BLASTP [29], [30] searches among the five genomes with an e-value cutoff of 1e^−15^. This choice of a stringent e-value cutoff prevents spurious hits between non-homologous genes that share some conserved domains and facilitates the identification of true homologous genes. The similarity results were supplied as the input for OrthoMCL [38] to perform homologous gene clustering. The algorithm is largely based on the popular criterion of reciprocal best hits between genomes for the identification of orthologous genes and includes additional normalization steps for between- and within-genome comparisons; an independent benchmarking study [39] has confirmed the reliability of this algorithm. For PnWB-specific genes, additional BLASTP [29], [30] searches against the NCBI nr db [31] were performed to identify putative homologous genes in other bacteria. All data parsing and processing steps were handled by a set of custom Perl scripts written with Bioperl modules [40].

To correct for possible over-annotation, the predicted protein-coding genes that were shorter than 100 amino acids, had a confidence score lower than 10 from the Prodigal [37] prediction, and lacked any functional predictions or putative homologous sequences were eliminated from the final annotation. To correct for possible under-annotation, protein sequences from the four complete phytoplasma genomes were used as queries to perform TBLASTN [29], [30] searches against the PnWB NTU2011 assembly.

To identify putative effectors, which were difficult to detect and annotate through sequence similarity searches, we adopted the method described in Bai et al. [19]. The program SignalP v4.0 [41] was used to predict the presence and location of signal peptide cleavage site in all protein sequences. The options were set to ‘-t gram+ -s notm’ to specify the use of Gram-positive bacteria model and noTM neural network. For proteins that contained a cleavage site between the first 20-51 a.a., we removed the N-terminal signal peptide and checked the presence of transmembrane domains by TMHMM v2.0 [42]. Proteins that contained a signal peptide but no transmembrane domain were considered as putative effectors in our annotation and comparative analysis.

For functional categorization, all protein sequences were annotated by utilizing the KAAS tool [43] provided by the KEGG database [44], [45] using the bi-directional best hit method. In addition to the default ‘Prokaryotes’ representative set, we included selected Tenericutes and Firmicutes in the reference genomes. The complete list of abbreviated genome ids selected includes: *hsa*, *dme*, *ath*, *sce*, *pfa*, *eco*, *sty*, *hin*, *pae*, *nme*, *hpy*, *rpr*, *mlo*, *bsu*, *sau*, *lla*, *spn*, *cac*, *mge*, *mtu*, *ctr*, *bbu*, *syn*, *aae*, *mja*, *afu*, *pho*, *ape*, *mpn*, *mmy*, *mmo*, *maa*, *uur*, *poy*, *acl*, *mfl*, *bha*, *bce*, *lca*, and *cbo*. The KEGG orthology assignments were further mapped to the COG functional category assignment [16], [17] to generate summary statistics (Figure 2). Genes that did not have any COG functional category assignment were assigned to a custom category (category X: no COG assignment). Finally, the annotation was manually curated to incorporate evidence from each of the approaches described above and to ensure the consistency of gene descriptions.

## Supporting Information

Table S1 Summary of protein-coding gene repertoire by functional categories. Summary of protein-coding gene repertoire in the five available phytoplasma genomes by functional categories. The table format is adopted from Kube et al. [12].
(XLS)

Table S2 List of homologous gene clusters. List of homologous gene clusters in the five available phytoplasma genomes.
(XLS)

Table S3 List of putative effectors. List of putative effectors in the five available phytoplasma genomes.
(XLS)

Table S4 List of PMU-associated genes. List of PMU-associated genes in the five available phytoplasma genomes. *Putative homologous genes that are not included in the same cluster as the PnWB gene by OrthoMCL and identified through additional BLASTP searches.
(XLS)
